# Alternate Service Delivery Models in Cancer Genetic Counseling: A Mini-Review

**DOI:** 10.3389/fonc.2016.00120

**Published:** 2016-05-13

**Authors:** Adam Hudson Buchanan, Alanna Kulchak Rahm, Janet L. Williams

**Affiliations:** ^1^Geisinger Health System, Genomic Medicine Institute, Danville, PA, USA

**Keywords:** genetic counseling, telemedicine, access, cancer, comparative effectiveness

## Abstract

Demand for cancer genetic counseling has grown rapidly in recent years as germline genomic information has become increasingly incorporated into cancer care, and the field has entered the public consciousness through high-profile celebrity publications. Increased demand and existing variability in the availability of trained cancer genetics clinicians place a priority on developing and evaluating alternate service delivery models for genetic counseling. This mini-review summarizes the state of science regarding service delivery models, such as telephone counseling, telegenetics, and group counseling. Research on comparative effectiveness of these models in traditional individual, in-person genetic counseling has been promising for improving access to care in a manner acceptable to patients. Yet, it has not fully evaluated the short- and long-term patient- and system-level outcomes that will help answer the question of whether these models achieve the same beneficial psychosocial and behavioral outcomes as traditional cancer genetic counseling. We propose a research agenda focused on comparative effectiveness of available service delivery models and how to match models to patients and practice settings. Only through this rigorous research can clinicians and systems find the optimal balance of clinical quality, ready and secure access to care, and financial sustainability. Such research will be integral to achieving the promise of genomic medicine in oncology.

## Introduction

The world of cancer genetics has experienced exponential growth in diagnostic and treatment opportunities that use genomic sequencing information, as was most recently acknowledged by the national Precision Medicine Initiative (1). Even before the Precision Medicine Initiative, however, demand for cancer genetic counseling grew as germline genetic testing became increasingly incorporated into breast and ovarian cancer treatment decisions (2, 3), public coverage of celebrity *BRCA* mutation status (4) reached a wide segment of the U.S. population (5), and multi-gene panels for hereditary cancer susceptibility were introduced (6, 7). Due to these factors, cancer genetics clinicians across the U.S. noted an increase in referrals for hereditary cancer risk assessment (8).

Cancer genetic counseling has traditionally been practiced in person, with patients traveling to a health-care facility to meet with a genetics clinician (9). The counseling process has typically involved at least two in-person visits – an initial visit to perform risk assessment and, if applicable, informed consent for genetic testing (“pretest counseling”) and for those who underwent genetic testing, a posttest visit to disclose test results and discuss results’ implications for cancer risk management in patient and family (10).

Pre- and posttest cancer genetic counseling is recognized to benefit individuals with cancer and their relatives. Counseling by clinicians trained in genetics has been associated with improved adherence to cancer risk management (11–14), better informed surgical decision making (2, 15), increased cancer genetics knowledge (16–19), high patient satisfaction (17, 20), and cost savings (21, 22). And, individuals who have undergone cancer genetic counseling, even those found to have a hereditary cancer syndrome, typically do not report long-term increased distress (18, 19, 23–30). Further, negative outcomes such as misinterpretation of test results, inappropriate medical management, and adverse psychosocial outcomes have been reported when genetic testing is performed without adequate genetic counseling (13, 21, 22, 31–34). In recognition of these benefits, pre- and posttest genetic counseling by qualified health professionals is recommended as standard-of-care by several professional organizations (35–39).

Yet, the confluence of new genomic sequencing techniques and greater public acceptance of cancer genetic counseling render the traditional in-person, multi-visit approach to genetic counseling insufficient to meet the demands of cancer genetics practice in the age of genomic medicine. Further, access to cancer genetics professionals varies widely across the U.S. (40–44). Rapid access to cancer genetic counselors is readily available in certain urban academic centers (45), but several groups, including rural residents, are underserved (9, 43, 44).

Alternate service delivery models for cancer genetic services have been proposed to improve access to care for individuals in underserved areas who are unable to travel to genetic counseling. The majority of genetic counselors report having used at least one alternate service delivery model (46). Here, we summarize the state of the science on alternate service delivery models for cancer genetic counseling and recommend future research on the effectiveness of these models. First, we present models in which genetics clinicians use alternate communication technologies to reach patients, followed by alternate visit models (group counseling and non-genetics clinician counseling) and direct-access testing models.

## Alternate Technology Models

### Pretest Telephone Counseling

Telephone counseling refers to pretest genetic counseling that is provided remotely by telephone (47). It has been used by a substantial minority of cancer genetic counselors (9). Randomized trials comparing telephone with in-person cancer genetic counseling have shown that telephone counseling achieves short-term outcomes as well as in-person counseling. These trials have shown no difference by group on patients’ knowledge (48, 49), psychosocial outcomes (e.g., distress, decisional conflict, and cancer worry) (48–50), satisfaction (50, 51), or patient-centered communication (49, 50). One study has shown cost savings to patients and institutions in telephone vs. in-person cancer genetic counseling (48). Among the outstanding research questions in telephone genetic counseling is whether telephone counseling facilitates psychosocial assessment and counseling to the same degree as in-person counseling, a concern raised in two studies (51, 52).

### Pretest Telephone Counseling and Educational Materials, Posttest In-Person Counseling

A Dutch group has tested a model, termed “DNA-Direct,” that uses a telephone consult plus mailed educational information for pretest counseling for hereditary breast and ovarian cancer syndrome, followed by in-person disclosure of genetic test results (53). In a non-randomized comparison of this model with traditional in-person pre- and posttest genetic counseling, the authors found favorable psychosocial outcomes in the DNA-Direct model, including lower distress and decisional conflict than in the traditional genetic counseling group (53, 54). Time to results disclosure was also lower in the DNA-Direct group.

### Posttest Telephone Counseling

By far, the most commonly used alternate service delivery model in the U.S. is telephone disclosure of genetic test results (i.e., a posttest phone visit), which typically follows an in-person pretest visit (46). Although disclosure of test results *via* phone is widely used by cancer genetic counselors, a minority of cancer genetic counselors report using the phone as the primary model for results disclosure (46, 55, 56). Genetic counselors who disclose results by phone appreciate the convenience it provides to patients (56) and the medical benefits of disclosing results to patients more quickly than in-person disclosure, facilitating more timely cancer risk management (55, 56). Still, some genetic counselors have reported being uncomfortable returning certain genetic test results by phone (e.g., mutation positive results) (56).

Telephone disclosure of genetic testing results has been shown to be acceptable to patients. A randomized comparison of phone vs. in-person disclosure of results showed no difference by group in anxiety, distress, cancer genetics knowledge, or patient satisfaction (57). Further, this study found that a significantly higher proportion of participants in the in-person group would have preferred phone disclosure, compared with the proportion of phone disclosure participants who would have preferred in-person disclosure (57). Retrospective, non-randomized studies of method of results disclosure have found no difference by group (phone vs. in-person) on patient outcomes such as cancer worry, cancer risk perception, patient satisfaction, or cancer risk management behaviors (e.g., surveillance, prophylactic surgery) (55, 58). Of note, patient satisfaction with the model of results disclosure was significantly higher when patients were allowed to choose the model (55).

### Telegenetics

Telegenetics is genetic counseling provided remotely by live videoconferencing, with visual and audio access (47). It has been most studied in the context of pretest cancer genetic counseling, but has been used for posttest counseling, too. Typically, the approach consists of a genetics clinician at an urban health-care facility seeing a patient who has come to a different, often rural, healthcare facility. It has been used by a substantial minority of cancer genetic counselors (9, 46, 59), but is rarely the sole service delivery model used by a counselor (9). Patients have reported high satisfaction with telegenetics (60–65) due to convenience (63) and savings in cost and time (62).

However, comparative effectiveness research on telegenetics is limited. Our randomized trial of telegenetics vs. in-person cancer genetic counseling found that telegenetics is substantially cheaper for institutions than in-person counseling, with no difference in patient satisfaction by group (65). But, while early reports show that telegenetics may facilitate psychosocial assessment and counseling (64), neither behavioral outcomes (e.g., adherence to recommended cancer risk management) nor psychosocial outcomes of cancer telegenetics have been assessed in randomized trials (60). Further, data are mixed on whether telegenetics actually improves access to care (60). Cohen et al. found that telegenetics was used most for patients who lived more than 2 h away from the genetics center, but did not find that telegenetics was associated with shorter wait times to an appointment than in-person counseling (9). Finally, attendance of cancer genetic counseling was lower in the telegenetics than in-person group in our randomized trial, indicating that telegenetics may not be acceptable to all patients (65).

## Alternate Visit Models

### Group Counseling

Group counseling occurs when multiple individuals have pretest genetic counseling together, typically for the same indication (e.g., all have a family history of breast cancer) (47). Group counseling can be performed *via* multiple communication technologies, though it is typically performed in person. Depending on the study, patients may have the opportunity for individual discussions of personal issues with a genetics clinician immediately after the group session (16, 66) or *via* a subsequent telephone consult (67). Group genetic counseling has been used by up to 10% of cancer genetic counselors, but is rarely the sole service delivery model used by a counselor (9, 46).

Group genetic counseling has shown promise for increasing efficiency by decreasing per-patient time for genetics clinicians (16, 67). And, a randomized comparison of group vs. individual cancer genetic counseling showed no difference by group in cancer-specific distress or knowledge of breast cancer genetics (16). Similarly, a non-randomized comparison of group vs. individual cancer genetic counseling showed no difference in perceived personal control, cancer-specific distress, or patient satisfaction (66). However, questions remain about whether group counseling would be widely accepted by cancer genetic counseling patients. One study showed a high rate of declining group counseling, concerns about the effects of group dynamics on patients’ privacy and decision making, and a preference for individual counseling over group counseling (67). A later, non-randomized study echoed this preference for individual counseling when patients were given the choice of service delivery model (66). This study also showed a lower rate of genetic testing uptake in the group counseling cohort than in the individual counseling cohort, though it is unclear whether this was due to a difference by cohort in the proportion of individuals for whom genetic testing was indicated or to a difference by cohort in the informed consent process (66). Further, it is not clear whether group genetic counseling improves access to care in underserved areas (9). And, as with other service delivery models, reimbursement for group counseling remains a challenge (9).

### Non-Genetics Clinician Counseling

Several additional models in which a non-genetics clinician is the primary provider of genetic counseling have been described. These include models in which non-genetics clinicians provide pretest counseling and refer either all patients to a genetics clinician posttest or just patients considered complex; genetic counselors assist non-genetics clinicians in risk assessment and pre- and posttest counseling, and may see some complex cases themselves; and a genetic counselor educates a community of clinicians on pre- and posttest counseling and trains them to manage routine cases and refer complex cases (46). Although these models appear to be fairly widely used, with up to 36% of genetic counselors having been involved in one of these models (46), data on the comparative effectiveness of these models for improving access to care or facilitating the same beneficial behavioral and psychosocial outcomes as two-visit, in-person genetic counseling by trained genetics clinicians is lacking.

## Direct-Access Genetic Testing

Also known as direct-to-consumer testing, direct-access testing occurs when individuals order their own genetic testing from a commercial laboratory outside the context of a typical medical encounter and receive results and associated educational materials directly. Although much of the available direct-access testing focuses on genomic variants with a modest impact on cancer risk, some tests do report mutations in genes associated with hereditary cancer syndromes (e.g., *BRCA1/2*). It is also possible for patients to initiate testing for hereditary cancer syndromes through companies that coordinate with their physicians and provide access to genetic counseling. Data on patient outcomes of direct-access testing for hereditary cancer syndromes are limited, with case reports showing both benefits of this approach as a way to be tested without concern for genetic discrimination (68) and concerns about increased psychological stress when a *BRCA* mutation is detected incidentally *via* direct-access testing (69). Preliminary qualitative research suggests that initial negative psychological outcomes of direct-access identification of hereditary cancer risk may be temporary (70). As direct-access models grow in prevalence, comparative effectiveness studies with traditional genetic counseling models will become necessary.

## Discussion

Alternate service delivery models have the potential for improving access to cancer genetic counseling, which is of growing importance as germline genomic information is increasingly incorporated into the care of individuals with cancer and their at-risk relatives. Such improved access may mitigate health disparities and help achieve the significant promise of genomic medicine (71). Telephone counseling, group counseling, and telegenetics have been well accepted by patients and may facilitate the patient-centered communication and psychosocial assessment that are the hallmark of cancer genetic counseling (16, 48, 51, 64, 65). Yet, considerable comparative effectiveness research is necessary to determine whether alternate service delivery models are as beneficial as in-person cancer genetic counseling. This holds true for alternate service delivery models provided by genetics and non-genetics clinicians. The latter is particularly important, given reports of negative outcomes of non-genetics clinicians providing cancer genetic counseling (13, 21, 22, 32–34).

Further studies are needed on the degree to which alternate service delivery models improve access to cancer genetic counseling. And here, we mean access broadly defined, not simply a patient’s ability to be seen with limited disruption of their daily responsibilities, though this is important. Ideally, access would mean that patients can have cancer genetic counseling that is readily available, affordable, and comparable to in-person counseling on outcomes of import to patients, genetics clinicians, and referring clinicians. Determining whether an alternate service model (or suite of models) is comparable to in-person counseling will require rigorous methodology and a focus on patient-centered outcomes such as longer-term psychosocial outcomes and adherence to recommended cancer risk management (60, 65). Studies conducted with a cost analysis that includes real-world reimbursement of genetic counseling – a significant challenge to broad implementation of all alternate service delivery models (9, 72, 73) – will also be critical.

One additional lesson of comparisons of alternate service delivery models with traditional in-person cancer genetic counseling is that one size will not fit all. Uptake of cancer genetic counseling has differed by service delivery model (48, 53, 65–67), and patients may be most satisfied when they are allowed to choose the method in which they have genetic counseling (55). This suggests a pragmatic research agenda that helps match service delivery models to patients and practice settings. Such research should investigate the wide variety of patient characteristics that could impact their preference for a particular service delivery model [e.g., cancer status (affected vs. unaffected), demographic characteristics, comfort with technology, and distance to the nearest genetics facility]. Clinically, the lesson that one size will not fit all suggests that cancer genetic counseling patients will be best served by being presented with a variety of service delivery models and allowed to choose their preferred model.

Using alternate service delivery models to provide cancer genetic counseling involves balancing several factors thought to be important to the clinical experience, including patients’ access to care and clinicians’ perceptions of their own effectiveness to clearly explain potentially complex genetics concepts while assessing and responding to psychosocial cues. And, models ultimately need to strike this balance while maintaining patients’ confidentiality, fitting into healthcare systems’ work flows, and being financially viable. Telegenetics, which facilitates an educational and empathetic interaction quite similar to an in-person conversation (63), holds promise for meeting the clinical rigor genetics clinicians expect. And, several videoconferencing programs have the necessary security protocols to maintain confidentiality. But, studies of telegenetics to date have focused on a model in which patients must attend a local health-care facility, potentially limiting some patients’ access and requiring staff at the remote clinic to facilitate patients’ interaction with the genetics clinician. With U.S. Internet use approaching 90% (74) and the proliferation of smartphones (75), telegenetics sessions that meet patients where they are on their preferred device may provide an even better balance of rapid access and high-quality care that has a minimal impact on clinics’ work flows. Whether such a model would be financially viable or help genetics clinicians meet growing demand for their services, however, remains to be seen.

## Author Contributions

AB: substantial contribution to conception of the work; drafting the work; final approval of version to be published; and agreement to be accountable for all aspects of the work. AR and JW: substantial contribution to conception of the work; revising the work for important intellectual content; final approval of version to be published; and agreement to be accountable for all aspects of the work.

## Conflict of Interest Statement

The authors declare that the preparation of this mini-review was conducted in the absence of any commercial or financial relationships that could be construed as a potential conflict of interest.
